# Psychological Symptoms and Behavioral Changes in Children and Adolescents During the Early Phase of COVID-19 Quarantine in Three European Countries

**DOI:** 10.3389/fpsyt.2020.570164

**Published:** 2020-12-03

**Authors:** Rita Francisco, Marta Pedro, Elisa Delvecchio, Jose Pedro Espada, Alexandra Morales, Claudia Mazzeschi, Mireia Orgilés

**Affiliations:** ^1^Católica Research Centre for Psychological - Family and Social Wellbeing, School of Human Sciences, Universidade Católica Portuguesa, Lisbon, Portugal; ^2^Department of Philosophy, Social Sciences and Education, Università degli Studi di Perugia, Perugia, Italy; ^3^Health Psychology Department, Universidad Miguel Hernández, Elche, Spain

**Keywords:** COVID-19, quarantine, psychological symptoms, behavioral symptoms, child habits, housing conditions, children, adolescents

## Abstract

The coronavirus disease (COVID-19) pandemic forced the home confinement of the majority of population around the world, including a significant number of children and adolescents, for several weeks in 2020. Negative psychological effects have been identified in adults, but research about the impact of this type of social distancing measure on children and adolescents is scarce. The present study aimed to describe and compare the immediate psychological and behavioral symptoms associated with COVID-19 quarantine in children and adolescents from three southern European countries with different levels of restrictions (Italy, Spain, and Portugal). Parents of 1,480 children and adolescents (52.8% boys) between 3 and 18 years old (M = 9.15, SD = 4.27) participated in the study. An online survey using snowball sampling techniques was conducted during 15 days between March and April 2020, representing the early phase of the quarantine associated with COVID-19 outbreak. Parents answered questionnaires about sociodemographic data, housing conditions, immediate psychological responses during quarantine (e.g., anxiety, mood, sleep, and behavioral alterations), patterns of use of screens, daily physical activity, and sleep hours before and during the quarantine. The results revealed an increase in children's psychological and behavioral symptoms, increased screen-time, reduced physical activity, and more sleep hours/night. Italian children presented less psychological and behavioral symptoms compared with Portuguese and Spanish children. In general, hierarchical multiple regressions revealed that having an outdoor exit in the house (e.g., garden, terrace) contributed to lower levels of psychological and behavioral symptomatology. Future studies are needed to identify family and individual variables that can better predict children and adolescents' well-being during and after quarantine. Recommendations for families and implications for practice are discussed.

## Introduction

Evidence regarding experiences from past outbreaks reveals that quarantine can create a substantial strain on the population and create mental health problems [e.g., (1, 2)]. However, most studies have been focused on adult populations and the psychological impact of quarantine on children remains unclear. The worldwide coronavirus disease (COVID-19) pandemic has caused several governments in Europe to determine quarantine and home confinement. However, restrictions have been quite diverse across different European countries, and the impact of these differences on young people's mental health remains unknown. This present public health crisis and the risk of second-wave outbreaks make it urgent to investigate the psychological effects of this type of social distancing measure on children and adolescents, taking into account the different levels of restrictions established in three European countries: Italy, Spain, and Portugal.

Quarantine is a public health measure that includes the restriction of activities or the separation of healthy individuals who may have been exposed to an infectious agent or disease with the aim of monitoring symptoms and ensuring the early identification of cases (3). Throughout Europe, the severity and timing of restriction measures has differed from country to country. The point at which social distancing was enforced occurred at different dates in each country based on the infection curve. Italy, the first European Union nation to put its entire population under quarantine, closed schools on 5 March (24 February in Lombardy), 16 days after the first 50 cases of COVID-19 were reported. On the other hand, Spain and Portugal closed schools on 13 March, 4 and 12 days after the first 50 cases, respectively.

Another important difference pertains the quarantine status. In general, compliance with quarantine may be voluntary or ensured by governmental orders. Italy and Spain ordered mandatory quarantine, whereas Portugal ordered voluntary quarantine (more specifically, a “general duty of home confinement”). More importantly, no studies to date have examined whether mandatory vs. voluntary quarantine has differential effects on psychological outcomes. However, it has been suggested, for example, that the perception that others may benefit from one's situation can help to endure stressful situations and this might be the case for home-based quarantine (2).

Although the employment of lockdown practices is highly necessary to control the spread of COVID-19, public health organizations worldwide have noted the importance of supporting mental and psychosocial well-being during social isolation and restriction of movements imposed to people during quarantine (4). Consistent with these recommendations, studies from previous epidemics and recent research on COVID-19 unequivocally demonstrate the negative psychological impact of quarantine and home confinement in the general population [e.g., (2, 5–8)]. A recent review of evidence indicates several negative psychological effects associated with quarantine, including posttraumatic stress, depression, anxiety, confusion, and anger (2). This review also suggests that quarantine and home confinement may also have long-lasting psychological effects. Other studies report emotional reactions to social distancing such as fear, isolation, loneliness, and insomnia, highlighting boredom as the greatest emotional disincentive to compliance with quarantine (1).

Experience with previous quarantine outbreaks highlights the results of prolonged quarantine periods in aspects that may indirectly influence mental health, including loss of income during quarantine, loss of job after quarantine, and disturbances in family relationships (1, 9). Some have argued that this type of disease-containment measures could cause tensions within households, inhibiting family rituals, norms, and values, that may contribute to regulate family functioning in times of crisis (1, 10).

Public health organizations and mental health experts have also acknowledged the potential adverse psychological effects of quarantine on children and adolescents [e.g., (11–13)]. Children have unique and specific needs that are disturbed by COVID-19 quarantine that includes not only home confinement but the inability to go to school and interact with peers and teachers (14). However, significantly less evidence exists regarding the psychological impact of quarantine in children and adolescents. One of the few studies concerning this population (10) found that children who had been quarantined exhibited increased rates of posttraumatic stress symptoms compared with children who were not quarantined, indicating that home confinement can be traumatizing to a significant number of children. A recent study conducted in China in February 2020 with children aged 3–18 years concluded that the most frequent psychological and behavioral problems included clinginess, distraction, irritability, and fear of asking questions about the outbreak (12).

Some authors have suggested that stressors such as lengthy confinement, fear of infection, boredom, inadequate information, lack of contact with peers and significant others, and family economic stress, can have even more significant and enduring effects on young people. The lack of personal space at home and other housing conditions can also have a significant impact on the mental health of children and parents based on previous evidence (15). For example, housing conditions, such as small apartments with limited views and indoor qualities, were related to depressive symptoms in a recent original study that investigated the effects of housing environment characteristics on mental health during the COVID-19 lockdown using a large sample of Italian university students (16). In addition, psychosocial stress and lifestyle alterations caused by home confinement could exacerbate the negative consequences on a child's physical and mental health, which may create a vicious circle (12, 17). Children's particular vulnerabilities to trauma (18), adverse events (12), and environmental risks (17) make them an important group to study the negative psychological effects of COVID-19 quarantine. Despite its importance, this topic has been somehow neglected in the literature and young people's reactions during epidemics remains understudied. This is unfortunate given that understanding young people's behaviors and emotions is essential to (1) accurately address their needs and (2) develop contextually relevant material and preventive actions for children and adolescents that may help to protect their mental health during quarantine measures. The abovementioned research indicates that the psychological effect of quarantine can be long lasting (2), emphasizing the need to ensure that effective mitigation measures are developed as part of the quarantine planning process. Therefore, there is a global and urgent responsibility of parents and governments to guarantee that children and adolescents are protected from the psychological and physical impact of COVID-19 quarantine (17).

### The Current Study

This study aims to compare immediate psychological effects of COVID-19 quarantine in children and adolescents from three southern European countries with different levels of restrictions: Italy, Spain, and Portugal. The specific objectives are (a) to identify differences in sociodemographic variables and housing conditions across countries; (b) to describe child immediate psychological and behavioral alterations (anxiety, mood, sleep, behavioral, feeding, and cognitive alterations) during COVID-19 quarantine and compare across countries; (c) to describe child habits (use of screens, daily physical activity, and hours of sleep) before and after quarantine and explore differences across countries; and (d) to identify explanatory factors for the psychological and behavioral alterations during quarantine by considering housing conditions.

## Materials and Methods

### Participants

Parents (M = 42.26 years, SD = 5.92) of a total of 1,480 children and adolescents between 3 and 18 years old (M = 9.15, SD = 4.27) from Italy (*n* = 712 from 94 cities), Spain (*n* = 431 from 84 cities), and Portugal (*n* = 335 from 94 cities) participated in the study. The majority of respondents were women (87.8%) and reported a monthly family income between 2,000 and 2,999 euros (31.8%). Table 1 presents the characteristics of the sample and its equivalence by country. No differences were observed across countries with regard to parents' gender and age, monthly family income, and children' gender. Spanish children are younger than Italian children (but with a small effect).

**Table 1:** Sample characteristics and equivalence by country.

	**Total (*n* = 1,480)**	**Italy (*n* = 712)**	**Spain (*n* = 431)**	**Portugal (*n* = 335)**	**Test^a^**	**Effect size^b^**
Parents						
Female [*N* (%)]	1,299 (87.8)	627 (88.1)	379 (87.9)	293 (86.9)	0.28	–
Age [M (SD)]	42.26 (5.92)	42.38 (6.64)	42.17 (5.32)	42.10 (4.96)	2.68	–
Monthly family income (euros)						
Up to 999	87 (6.6)	33 (5.3)	31 (8.3)	23 (7.3)	14.82	–
Between 1,000 and 1,999	372 (28.2)	164 (26.2)	113 (30.1)	95 (30.1)		
Between 2,000 and 2,999	417 (31.8)	209 (33.4)	98 (26.1)	110 (34.8)		
Between 3,000 and 4,999	343 (26)	169 (27)	106 (28.3)	68 (21.5)		
5,000 or more	98 (7.4)	51 (8.1)	27 (7.2)	20 (6.3)		
The house where you live has [*N* (%)]						
Only windows	158 (10.7)	25 (3.5)	77 (17.9)	56 (16.6)	221.39***	0.27
Garden	559 (37.8)	368 (51.7)	77 (17.9)	114 (33.8)		
Terrace	303 (20.5)	151 (21.1)	121 (28.1)	31 (9.2)		
Balcony	416 (28)	141 (19.9)	145 (33.5)	130 (38.6)		
Another exit	44 (3)	27 (3.8)	11 (2.6)	6 (1.8)		
People who live in my house during quarantine [*N* (%)]						
They do not leave the house unless they • have to buy groceries or other allowed activities	936 (63.1)	463 (65)	254 (58.9)	217 (64.4)	4.59	–
One or both parents still work outside the home	546 (36.9)	249 (35)	177 (41.1)	120 (35.6)		
How many people live in at home during quarantine [M (SD)]	3.94 (0.94)	3.99 (0.97)	3.84 (0.88)	3.98 (0.95)	9.73**	0.007
Square meters home [M (SD)]	131.04 (67.70)	123.14 (62.29)	124.99 (62.86)	152 (78.89)	46.80***	0.03
Children						
Female [*N* (%)]	699 (47.2)	351 (49.3)	192 (44.5)	156 (46.3)	2.58	–
Age [M (SD)]	9.15 (4.27)	9.40 (4.46)	8.55 (3.73)	9.42 (4.45)	8.58*	0.006

*M, mean; SD, standard deviation*.

a *Cross-table (χ^2^) for categorical variables and Kruskal-Wallis (χ^2^) for continuous variables*.

b *Cramer's V for multi-categorical variables and Epsilon-squared for continuous variables*.

Significant differences (large effect) on housing conditions were reported between participants from the three countries. Portuguese homes are larger (more square meters) than Italian or Spanish homes. Regarding exits to the outside, Italian houses more frequently have gardens than Spanish houses, and houses with a terrace are more frequently found in Spain compared with Portugal. Houses with only windows or a balcony are more frequently noted in Spain and Portugal compared with Italy. Greater than half of the participants (58.2%, *n* = 862) had an outdoor exit. In general, most of the Italian children live in houses with a garden (51.7%), and most Spanish and Portuguese children have a balcony (33.5 and 38.6%, respectively).

The number of people living at home during quarantine is significantly lower in Spain compared with Italy and to Portugal. The frequency of people who do not leave the house (unless they have to buy groceries or other allowed activities) or who still work outside the home is not different among the three countries.

### Procedure

A cross-sectional design was used to assess the psychological symptoms and behavioral changes in children and adolescents during the early phase of the quarantine associated with COVID-19 from parents' perspective. Participants were recruited via social networks, including social media platforms (Facebook, LinkedIn, Instagram) and researchers' acquaintances (e-mail), using a snowball sampling strategy. An online survey was created *ad-hoc* and distributed in each country (via Qualtrics or GoogleForms) and data were collected for 15 days between March and April 2020. Before completing the survey, information about the objectives of the study was provided, and informed consent was requested. Each participant took ~10 min to complete the survey, and no compensation was provided. The study was approved by the Ethics Committees of the authors' institutions.

### Measures

The survey was constructed initially in English and *ad-hoc* for this study and included multiple choice and rating scale questions. The final version was pilot-tested by 10 families with children aged 3–18 years per country. Comprehension was adequate, and no changes were required in the survey.

A general questionnaire included sociodemographic questions (e.g., participant age and gender, marital status, family income, and number of children) and questions about housing conditions (e.g., square meters and outdoor exits) and specifics about the period of quarantine (e.g., number of people living in at home during quarantine).

The questionnaire about children's immediate psychological responses during quarantine (“During the past few days, compared to before quarantine, to what extent have you noticed that your child…”) included 10 items related to “anxiety” (e.g., “is worried” and “is afraid of COVID-19 infection”), 6 items related to “mood” (e.g., “is sad”), 5 items related to “sleep” (e.g., “is afraid to sleep alone”), 6 items related to “behavioral alterations” (e.g., “argues with the rest of the family”), 2 items related to “feeding” (e.g., “eats a lot”), and 2 items related to “cognitive alterations” (e.g., “has difficulty concentrating”). Each item had two possible responses (yes or no). Ordinal alpha in the current sample is excellent (α = 0.96). Evidences of validity were found between the subscales and measures of anxiety (SCAS-P) and depression (SMFQ-P). Moderate correlations were observed between depression (SMFQ-P) and mood (ρ = 0.39) as well as between anxiety (SCAS-P) and anxiety (ρ = 0.46).

The questionnaire about children' habits included items about the patterns of use of screens (e.g., “Before quarantine, how long did your child use screens such as iPads, TVs, mobiles, or computers daily?”) and daily physical activity (e.g., “During quarantine, how much time did your child spend daily on physical activity?”) before and during the quarantine with six answer options (from “<30 min” to “more than 180 min”). Parents were also asked about the number of hours their children sleep during the weekdays (before and during the quarantine).

### Data Analyses

All calculations were performed using SPSS 26 for Mac. The Kolmogorov-Smirnov test was used to assess the normality of the data. Given the lack of normality in the continuous variables, non-parametric tests were used. Ordinal alpha, which is considered the most appropriate for ordinal items is calculated. Kruskal-Wallis tests were performed to compare continuous variables across countries (Italy, Spain, and Portugal) and Chi-squared tests were used to compare proportions across these groups. Differences were considered statistically significant when the *p* < 0.05. Bonferroni corrections applied to *p-*values were used to reduce the risk of type I errors *post-hoc* analysis of Chi-squared tests. In cross-tables, Chi-square *post-hoc* tests using adjusted residuals were calculated (19). Epsilon-squared (ε^2^) was used as an effect size, where small effect sizes ranged from 0.01 to <0.08, medium effect sizes ranged from 0.08 to <0.26, and large effect sizes ranged from ≥0.26. Cramer's *V* was calculated as a measure of association between multicategorical variables, and interpreted as follows: >0.25 very strong, >0.15 strong, >0.10 moderate, >0.05 weak, and > 0 none or very weak (20).

The Wilcoxon matched-pair signed rank test was used to evaluate change in patterns of use of screens, daily physical activity, and hours of sleep before and during the quarantine within a group. The effect size of the statistically significant differences was estimated using Rosenthal's *r*, which is interpreted as follows: 0.10 = small, 0.30 = medium, and 0.50 = large (21). Mann-Whitney *U*-test was performed to analyze the relationship between outdoor exit (yes/no) and main outcomes.

Spearman correlations were calculated to analyze the relationship between continuous variables included in the hierarchical regression analyses. To test the association between having more symptoms in these six areas (“anxiety,” “mood,” “sleep,” “behavioral alterations,” “feeding,” and “cognitive alterations”) during quarantine and housing conditions, six separate hierarchical regression analyses were run using anxiety, mood, sleep, behavioral alterations, feeding, and cognitive alterations as dependent variables. Children's sex and age were included in a first step as covariates. Outdoor exit (yes/no), number of people living at home during quarantine, and square meters home were included in the second step as independent variables. Having a garden or terrace was recoded as 1 (= outdoor exit). Options “only windows,” “balcony,” and “other exits” were coded as 0 (no outdoor exit). Interactions between variables were analyzed in step 3. Continuous variables were mean centered to avoid multicollinearity.

## Results

The present study aimed to compare immediate psychological and habit changes during COVID-19 quarantine in children and adolescents from Italy, Spain, and Portugal. We also intended to identify explanatory factors for the psychological and behavioral symptoms based on housing conditions.

### Immediate Psychological and Behavioral Symptoms in Children During Quarantine Across Countries

Table 2 presents parents' perception of their children's anxiety, mood, sleep, behavioral, feeding, and cognitive alterations during quarantine and differences across countries. *Post-hoc* analysis revealed significant differences across countries in all dimensions in total scores (small effects) and in almost all specific items that contribute to each dimension (with small and medium effects), which are detailed in Table 2.

**Table 2:** Differences across countries in children's psychological and behavioral symptoms during quarantine (parents' perceptions).

	**Total (*****n*** **= 1,480)**		**Italy (1) (*****n*** **= 712)**		**Spain (2) (*****n*** **= 431)**		**Portugal (3) (*****n*** **= 335)**		**Test^a^**	**Effect size^b^**	***Post-hoc***
	***N***	**%**	***n***	**%**	***n***	**%**	***n***	**%**			
**Anxiety/Activation**											
My child is worried	495	33.4	226	31.7	118	27.4	151	44.8	27.59^***^	0.13	3>2
My child is anxious	446	30.1	146	20.5	179	15.7	121	35.9	63.27^***^	0.20	3 > 1; 3 > 2
My child is nervous	543	36.7	243	34.1	191	44.3	109	32.3	15.54^***^	0.10	2 > 1; 2 > 3
My child worries when one of us leaves the house	350	23.6	121	17	130	30.2	99	29.4	33.71^***^	0.15	2 > 1; 3 > 1
My child is restless	563	38	247	34.7	196	45.5	120	35.6	1434^**^	0.10	3 > 2; 1 > 2
My child is afraid of COVID-19 infection	403	27.2	164	23	100	23.2	139	41.2	43.26^***^	0.17	3 > 1; 3 > 2
My child is uneasy	501	33.9	184	25.8	163	37.8	154	45.7	44.54^***^	0.17	3 > 1
My child is easily alarmed	214	14.5	78	11	60	13.9	76	22.6	25.01^***^	0.13	3 > 1
My child has physical complaints (headache, stomach ache,.)	193	13	72	10.1	87	20.2	34	10.1	27.37^***^	0.13	2 > 1; 2 > 3
My child asks about death	202	13.6	102	14.3	53	12.3	47	13.9	0.97	–	–
Anxiety total [M (SD), range = 0–10]	2.64	2.53	2.22	2.38	2.96	2.62	3.11	2.58	40.96^***^	0.02	2 > 1; 3 > 1
**Mood**											
My child is sad	351	23.7	189	26.5	77	17.9	85	25.2	11.71^**^	0.09	1 > 2; 3 > 2
My child is reluctant	345	23.3	192	27	90	20.9	63	18.7	10.76^**^	0.08	1 > 2; 1 > 3
My child feels lonely	491	33.2	280	39.3	78	18.1	133	39.5	62.36^***^	0.20	1 > 2; 3 > 2
My child cries easily	261	17.6	97	13.6	98	22.7	66	19.6	16.49^***^	0.10	2 > 1
My child feels frustrated	328	22.2	113	15.9	100	23.2	115	34.1	44.53^***^	0.17	3 > 1
My child is bored	772	52.2	383	53.8	213	49.4	176	52.2	2.05	–	–
Mood total (M (SD), range = 0–6)	1.72	1.61	1.76	1.62	1.52	1.50	1.89	1.70	8.71^*^	0.006	3 > 2
**Sleep**											
My child wakes up frequently	180	12.2	70	9.8	68	15.8	42	12.5	8.92^*^	0.08	2 > 1
My child sleeps little	189	12.8	52	7.3	31.3	16.9	64	19	37.52^***^	0.16	3 > 1; 3 > 2
My child is afraid to sleep alone	253	17.1	94	13.2	103	23.9	56	16.6	21.74^***^	0.12	2 > 1
My child has nightmares	169	11.4	62	8.7	64	14.8	43	12.8	10.78^**^	0.08	2 > 1
My child has sleeping difficulties	249	16.8	90	12.6	105	24.3	54	16.02	26.56^***^	0.13	2 > 1
Sleep total (M (SD), range = 0–5)	0.70	1.21	0.51	1.06	0.95	31.8	0.76	1.21	42.73^***^	0.02	3 > 1; 2 > 1
**Behavioral alterations**											
My child argues with the rest of the family	447	30.2	165	23.2	174	40.4	108	32	38.36^***^	0.16	2 > 1
My child is irritable	598	40.4	260	36.5	186	43.2	152	45.1	8.91^*^	0.07	2 > 1; 3 > 1
My child has behavioral problems	246	16.6	57	8	128	29.7	61	18.1	91.85^***^	0.25	2 > 1
My child is angry	388	26.2	157	22.1	139	32.3	92	27.3	14.70^**^	0.10	2 > 1
My child is very quiet	159	10.7	102	14.3	24	5.6	33	9.8	21.88^***^	0.12	1 > 2
My child is very dependent on us	394	26.6	163	22.9	157	36.4	74	21.9	30.03^***^	0.14	2 > 1
Behavioral alterations total (M (SD), range = 0–6)	1.50	1.61	1.26	1.38	1.87	1.82	1.54	1.68	23.93^***^	0.01	2 > 1; 2 > 3
**Feeding**											
My child eats a lot	343	23.2	142	19.9	108	25.1	93	27.6	8.73^*^	0.07	2 > 1; 3 > 1
My child has no appetite	138	9.3	48	6.7	50	11.6	40	11.9	10.84^**^	0.08	2 > 1; 3 > 1
Feeding total (M (SD), range = 0–2)	0.32	0.54	0.26	0.49	0.36	0.54	0.39	0.62	14.90^**^	0.01	3 > 1; 2 > 1
**Cognitive alterations**											
My child is very indecisive	173	11.7	62	8.7	69	16	42	12.5	14.11^**^	0.10	2 > 1
My child has difficulty concentrating	353	23.9	135	18.9	133	30.85	85	25.2	21.37^***^	0.12	2 > 1
Cognitive alterations total (M (SD), range = 0–2)	0.35	0.60	0.27	0.54	0.46	0.66	0.37	0.61	28.95^***^	0.02	3 > 1; 2 > 1

* *p < 0.05*;

** *p < 0.01*;

*** *p < 0.001*.

a *Cross-table (χ^2^) for categorical variables and Kruskal-Wallis (χ^2^) for continuous variables*.

b *Cramer's V for multi-categorical variables and Epsilon-squared for continuous variables. Bonferroni correction applied to p-values was used to reduce the risk of type I errors post-hoc analysis of a Chi-squared test*.

In general, children from Italy had significantly lower levels of anxiety, sleep, feeding, and cognitive alterations compared with children from Spain and Portugal. Portuguese children presented significantly more mood alterations than Spanish children. Spanish children presented significantly more behavioral alterations than both Italian and Portuguese children.

Of note, approximately one-third of children are restless, nervous, worried, uneasy, and anxious. Additionally, 27.2% are afraid of COVID-19 infection, and Portuguese children are significantly more afraid of infection compared with the other children. Greater than half of the samples are bored (52.2%) and 1/3 feel lonely, especially Portuguese and Italian children. Considering behavioral alterations, >40% of children are irritable (especially the Portuguese and Spanish), and ~1/3 argues with the rest of the family more than before home confinement.

### Children's Patterns of Use of Screens, Daily Physical Activity, and Hours of Sleep Before and During Quarantine

Significant differences were found before and during quarantine in all habits analyzed both in the total sample and in the samples of each country. Table 3 presents children's patterns of use of screens, daily physical activity, and hours of sleep during weekdays before and during quarantine and differences across countries.

**Table 3:** Children's patterns of use of screens, daily physical activity, and hours of sleep before and during the quarantine and differences across countries (parents' perceptions).

**Children's activity patterns**	**Total (*****n*** **= 1,480)**		**Italy (1) (*****n*** **= 712)**		**Spain (2) (*****n*** **= 431)**		**Portugal (3) (*****n*** **= 335)**		**Before quarantine**			**During quarantine**		
	**Before**	**During**	**Before**	**During**	**Before**	**During**	**Before**	**During**	**Test^a^**	**Effect size^b^**	**Pair-wise^b^**	**Test^a^**	**Effect size^b^**	***Post-hoc*^b^**
**Use of screens [min**, ***N*** **(%)]**														
<30	306 (20.7)	44 (3)	129 (18.1)	32 (4.5)	124 (28.8)	7 (1.6)	53 (15.7)	5 (1.5)	30.02^**^	0.10	2 > 1; 2 > 3	58.92^***^	0.14	1 > 2; 1 > 3
From 30 to 60	529 (35.7)	158 (10.7)	252 (35.4)	99 (13.9)	151 (35)	37 (8.6)	126 (37.4)	22 (6.5)						1 > 2; 1 > 3
From 60 to 90	347 (23.4)	247 (16.7)	173 (24.3)	135 (19)	89 (20.7)	73 (16.9)	85 (25.2)	39 (11.6)						
From 90 to 120	162 (11)	307 (20.7)	86 (12.1)	129 (18.1)	39 (9)	85 (19.7)	37 (11)	93 (27.5)						3 > 1; 3 > 2
From 120 to 180	85 (5.7)	278 (18.8)	47 (6.6)	101 (14.2)	16 (3.7)	104 (24.2)	22 (6.5)	73 (21.7)						2 > 1
More than 180	51 (3.5)	446 (30.1)	25 (3.5)	216 (30.3)	12 (2.8)	125 (29)	14 (4.2)	105 (31.2)						
**Physical activity [min/day**, ***N*** **(%)]**														
<30	189 (12.8)	785 (53)	125 (17.6)	404 (56.7)	30 (7)	231 (53.6)	34 (10.1)	150 (44.5)	56.43^***^	0.13	1 > 2	29.29^**^	0.09	1 > 3; 2 > 3
From 30 to 60	490 (33.1)	477 (32.2)	251 (35.2)	198 (27.8)	118 (27.4)	138 (32)	121 (36)	141 (41.8)						3 > 1
From 60 to 90	416 (28.1)	138 (9.3)	177 (24.9)	67 (9.4)	143 (33.2)	41 (9.5)	96 (28.5)	30 (8.9)						
From 90 to 120	198 (13.4)	50 (3.4)	83 (11.7)	27 (3.9)	67 (15.5)	11 (2.6)	48 (14.2)	12 (3.6)						
From 120 to 180	102 (6.9)	16 (1.1)	36 (5.1)	8 (1.1)	47 (10.9)	4 (0.9)	19 (5.6)	4 (1.2)			2 > 1; 2 > 3			
More than 180	85 (5.7)	14 (1)	40 (5.5)	8 (1.1)	26 (6)	6 (1.4)	19 (5.6)	0 (0)						
**Hours of sleep/week [M (SD)]**	9.11 (1.44)	9.51 (1.55)	8.86 (1.56)	9.22 (1.65)	9.44 (1.16)	9.66 (1.38)	9.23 (1.39)	9.91 (1.41)	91.14^***^	0.06	3 > 1; 2 > 1; 2 > 3	67.31^***^	0.04	2 > 1; 3 > 1

** *p < 0.01*;

*** *p < 0.001*.

a *Cross-table (χ^2^) for categorical variables and Kruskal-Wallis (χ^2^) for continuous variables*.

b *Cramer's V for multi-categorical variables and Epsilon-squared for continuous variables. Bonferroni correction applied to p-values was used to reduce the risk of type I errors post-hoc analysis of a Chi-squared test*.

Daily use of screens noticeably increased during quarantine (*z* = −30.34, *p* < 0.001, *r* = 0.78). Before quarantine, most children used screens from 30 to 60 min/day (35.7%), whereas the majority of children had more than 3 h of screen time during quarantine (30.1%). This pattern is roughly the same in all countries (Italy: *z* = −20.33, *p* < 0.001, *r* = 0.76; Spain: *z* = −16.91, *p* < 0.001, *r* = 0.81; Portugal: *z* = −14.90, *p* < 0.001, *r* = 0.79). Differences across countries (medium effect) before quarantine were found only for the use of screens for <30 min; specifically, Spanish children more frequently used screens <30 min/day. However, during quarantine, more differences were noted across countries (see Table 3 for detailed *post-hoc* analyses); for example, Italian children use screens less (<30 min or between 30 and 60 min), whereas Spanish children significantly use screens more frequently (between 120 and 180 min).

Regarding patterns of physical activity, large effects were found when comparing changes before and during quarantine, for all the sample (*z* = −25.56, *p* < 0.001, *r* = 0.66) and for each country in particular (Italy: *z* = −16.08, *p* < 0.001, *r* = 0.60; Spain: *z* = −15.45, *p* < 0.001, *r* = 0.74; Portugal: *z* = −12.48, *p* < 0.001, *r* = 0.66). Before quarantine, most children practiced 30 to 60 min of physical activity daily (33.1%). However, in quarantine, most children experienced <30 min of physical activity (53%). Significant differences across countries (medium effects) were also found (see Table 3). For example, before quarantine, Spanish children more frequently practiced physical activity between 120 and 180 min. During quarantine, Portuguese children less frequently participated in <30 min of physical activity.

The mean number of hours of sleep during weekdays significantly increased during home confinement for the total sample (*z* = −11.75, *p* < 0.001, *r* = 0.30) and for each country (Italy: *z* = −8.78, *p* < 0.001, *r* = 0.32; Spain: *z* = −3.02, *p* < 0.001, *r* = 0.14; Portugal: *z* = −8.74, *p* < 0.001, *r* = 0.46). Additionally, significant differences (small effects) were found across the countries. Italian children slept significantly less than Spanish and Portuguese children both before and during quarantine. Before quarantine (but not during this period), Spanish children slept more than Portuguese children.

### Housing Conditions and Children's Psychological and Behavioral Symptoms During Quarantine

Table 4 presents the descriptive statistics and correlations among children's age, housing conditions, and psychological and behavioral symptoms in study. The size of the home (square meters) was unrelated to the outcome variables. Fewer people at home during quarantine were significantly related to having more mood symptoms, and child's age was significantly related to less symptoms of sleep and behavioral alterations. Children who did not have an outdoor exit at home (garden or terrace) were significantly more likely to present anxiety (*U* = 249,292, *z* = −2.13, *p* < 0.05, *r* = 0.05), sleep (*U* = 234,875.50, *z* = −4.59, *p* < 0.001, *r* = 0.11), behavioral (*U* = 244,057, *z* = −2.85, *p* < 0.01, *r* = 0.07), feeding (*U* = 245,348, *z* = −3.28, *p* ≤ 0.001, *r* = 0.08), and cognitive alterations (*U* = 251,278.50, *z* = −2.35, *p* < 0.05, *r* = 0.06) during home confinement compared with those who had an outdoor exit. Compared with girls, boys presented significantly increased levels of anxiety (*U* = 97,292.50, *z* = −3.82, *p* < 0.001, *r* = 0.09), mood (*U* = 95,936.50, *z* = −4.11, *p* < 0.001, *r* = 0.10), sleep (*U* = 99,242, *z* = −4.02, *p* < 0.001, *r* = 0.10), behavioral (*U* = 98,791.50, *z* = −3.61, *p* < 0.001, *r* = 0.09), feeding (*U* = 108,639.50, *z* = −2.09, *p* < 0.05, *r* = 0.05), and cognitive alterations (*U* = 103,479, *z* = −3.30, *p* ≤ 0.001, *r* = 0.08).

**Table 4:** Means, standard deviations, and correlations with confidence intervals.

**Variable**	**M**	**SD**	**1**	**2**	**3**	**4**	**5**	**6**	**7**	**8**
1. Square meters home	131.04	67.70								
2. Number of people at home	3.94	0.94	0.28^**^ [0.23, 0.33]							
3. Child age	9.15	4.28	0.11^**^ [0.06, 0.16]	0.06^*^ [0.01, 0.11]						
4. Anxiety/Activation	2.64	2.53	0.02 [−0.03, 0.07]	−0.01 [−0.06, 0.04]	−0.00 [−0.06, 0.05]					
5. Mood	1.72	1.62	−0.01 [−0.07, 0.04]	−0.07^**^ [−0.12, −0.02]	−0.01 [−0.07, 0.04]	0.54^**^ [0.50, 0.58]				
6. Sleep	0.70	1.21	−0.01 [−0.06, 0.04]	−0.02 [−0.07, 0.03]	−0.17^**^ [−0.22, −0.12]	0.42^**^ [0.38, 0.46]	0.33^**^ [0.28, 0.37]			
7. Behavioral alterations	1.51	1.62	−0.02 [−0.07, 0.04]	−0.00 [−0.05, 0.05]	−0.12^**^ [−0.17, −0.07]	0.61^**^ [0.57, 0.64]	0.59^**^ [0.55, 0.62]	0.40^**^ [0.36, 0.45]		
8. Feeding	0.33	0.54	−0.02 [−0.08, 0.03]	−0.01 [−0.06, 0.04]	−0.01 [−0.06, 0.05]	0.23^**^ [0.18, 0.28]	0.19^**^[0.14, 0.24]	0.16^**^ [0.11, 0.21]	0.26^**^ [0.21, 0.31]	
9. Cognitive alterations	0.36	0.61	0.02 [−0.03, 0.07]	0.01 [−0.04, 0.06]	−0.02 [−0.07, 0.03]	0.47^**^ [0.43, 0.51]	0.44^**^ [0.40, 0.48]	0.32^**^ [0.27, 0.36]	0.50^**^ [0.46, 0.54]	0.19^**^ [0.14, 0.24]

*Values in square brackets indicate the 95% confidence interval for each correlation. The confidence interval is a plausible range of population correlations that could have caused the sample correlation (22)*.

*M, mean; SD, standard deviation*.

* *p < 0.05*;

** *p < 0.01*.

Hierarchical multiple regression was performed to examine whether housing conditions (outdoor exit and number of people at home) predicted children's psychological and behavioral symptoms during quarantine after controlling for the influence of children's age and sex. Because the square meters of the home did not have a significant correlation with psychological or behavior symptoms, it was not included in the models. As shown in Table 5, both the presence of an outdoor exit and the number of people at home were significant predictors of symptomatology. Specifically, having an outdoor exit at home (garden or terrace) was a significant predictor of lower levels of all symptoms, except mood alterations. This was the only significant predictor of lower levels of anxiety, sleep, and cognitive alterations. The number of people living at home was another housing condition with a significant contribution to children's symptoms, particularly mood alterations. These results indicate that the lesser the number of people at home the higher the levels of mood alterations. After controlling for the effects of child age and sex, housing conditions explained between 1.2 and 3.9% of variance. Finally, age and sex were also significant predictors of children's symptoms. To be male was a significant predictor of anxiety symptoms, feeding, and behavior alterations and to be younger was a significant predictor of behavior and sleep alterations.

**Table 5:** Results from hierarchical regression examining the association between psychological and behavioral symptoms during quarantine and housing conditions, controlling for children's sex and age.

**Predictor variables**	***B***	**95% CI (B)**	**β**	***t* statistic**	***p*-value**	**Δ*R*^**2**^**	**Adj.*R*^**2**^**
**DV: anxiety/activation**							
Step 1							
Constant	2.76	2.58, 2.94		30.56	<0.001	0.003	0.002
Child sex	−0.26	−0.52, −0.004	−0.05	1.99	0.04		
Step 2							
Constant	2.94	2.71, 3.17		24.82	<0.001	0.006	0.005
Child sex	−0.25	−0.51, 0.001	−0.05	−1.96	0.04		
Outdoor exit	−0.31	−0.57, −0.04	−0.06	−2.32	0.02		
**DV: mood**							
Step 1							
Constant	1.72	1.61, 1.83		29.79	<0.001	0	−0.001
Child sex	−0.004	−0.16, 0.16	−0.001	−0.04	0.96		
Step 2							
Constant	2.20	1.83, 2.57		11.82	<0.001	0.005	0.004
Child sex	−0.01	−0.18, 0.15	−0.005	−0.17	0.85		
Number of people at home	−0.12	−0.20, −0.03	−0.07	−2.71	0.007		
**DV: behavioral alterations**							
Step 1							
Constant	2.03	1.82, 2.24		19.14	<0.001	0.019	0.018
Child age	−0.04	−0.06, −0.02	−0.12	−4.76	<0.001		
Child sex	−0.21	−0.37, −0.04	−0.06	−2.54	0.01		
Step 2							
Constant	2.13	1.73, 2.53		10.42	<0.001	0.026	0.024
Child age	−0.04	−0.06, −0.02	−0.12	−4.76	<0.001		
Child sex	−0.21	−0.37, −0.04	−0.06	−2.53	0.01		
Outdoor exit	−0.27	−0.43, −0.10	−0.08	−3.24	0.001		
Step 3: interaction effects							
Constant	2.24	1.69, 2.78		8.04	<0.001	0.028	0.024
Child age * child sex	0.02	−0.03, 0.07	0.06	0.85	0.39		
Child age * outdoor exit	−0.03	−0.06, 0.010	−0.06	−1.45	0.14		
Child sex * outdoor exit	0.05	−0.27, 0.38	0.01	0.31	0.75		
**DV: sleep**							
Step 1							
Constant	1.14	0.98, 1.30		14.41	<0.001	0.030	0.028
Child age	−0.05	−0.06, −0.03	−0.17	−6.69	<0.001		
Child sex	0.009	−0.11, 0.13	0.004	0.14	0.88		
Step 2							
Constant	1.27	1.10, 1.44		14.71	<0.001	0.038	0.037
Child age	−0.05	−0.06, −0.03	−0.17	−6.70	<0.001		
Child sex	0.01	−0.11, 0.13	0.005	0.18	0.85		
Outdoor exit	−0.23	−0.35, −0.11	−0.09	−3.71	<0.001		
Step 3: interaction effects							
Constant	1.21	0.98, 1.44		10.37	<0.001	0.039	0.036
Child age * outdoor exit	−0.01	−0.04, 0.01	−0.03	−0.75	0.44		
**DV: feeding**							
Step 1							
Constant	0.35	0.32, 0.39		18.47	<0.001	0.004	0.004
Child sex	−0.07	−0.12, −0.01	−0.06	−2.52	0.01		
Step 2							
Constant	0.41	0.36, 0.46		16.25	<0.001	0.012	0.010
Child sex	−0.07	−0.12, −0.01	−0.06	−2.48	0.01		
Outdoor exit	−0.09	−0.15, −0.03	−0.08	−3.30	0.001		
Step 3: interaction effects							
Constant	0.40	0.34, 0.45		13.44	<0.001	0.012	0.010
Child sex * outdoor exit	−0.05	−0.16, 0.06	−0.04	−0.82	0.40		
**DV: cognitive alterations**							
Step 1							
Constant	0.37	0.33, 0.42		17.39	<0.001	0.002	0.001
Child sex	−0.04	−0.10, 0.01	−0.04	−1.49	0.13		
Step 2							
Constant	0.41	0.36, 0.47		14.68	<0.001	0.005	0.003
Child sex	−0.04	−0.10, 0.01	−0.04	−1.47	0.14		
Outdoor exit	−0.07	−0.13, −0.007	−0.06	−2.19	0.029		

*Categorical variables: child sex (0 = male/1 = female) and outdoor exit (0 = no/1 = yes)*.

*DV, dependent variable; B, unstandardized regression coefficient; CI, confidence interval; β, standardized regression coefficient; t, obtained t value for each predictor variable; p, probability; ΔR^2^, proportion of variance explained; Adj.R^2^, adjusted proportion of variance explained*.

## Discussion

Despite previous research demonstrating the psychological impact of the imposition of quarantine in past pandemics, few studies have investigated the negative effects on children's and adolescents' mental health. The present study aimed to identify and compare immediate psychological and behavioral effects of COVID-19 quarantine in children and adolescents from Italy, Spain, and Portugal.

### Child Habits and Psychological and Behavioral Alterations During COVID-19 Quarantine

According to parents' perceptions, greater than half of the children feel bored, 40% were irritable, and approximately one-third feels more lonely, restless, nervous, worried, anxious, and uneasy, compared with the period before quarantine. This increase in symptomatology was expected based on past evidence regarding children and adults who experienced previous quarantine outbreaks (2) and recent studies on adults from China during the actual COVID-19 pandemics [e.g., (23)]. Parents also reported that their children argue more with the rest of the family during home confinement. Evidence shows that quarantine has adverse psychological effects on adults' mental health, causing depression, stress, anger, and boredom (e.g., 1,2) and that confinement of people at home can produce tensions within households (1, 10). Considering that parents in COVID-19 quarantine may be particularly distressed, these results might reflect less parental emotional availability to support children, increasing inadequate parenting practices, such as hostility or inconsistent discipline (24, 25). Consequently, children's and adolescents' symptomatology may increase, as well as the probability of arguing with family members. These findings seem to highlight the concerns from international health organizations regarding the impact of COVID-19 quarantine on children and adolescents' mental health and family relationships (4, 26, 27).

Children from the three countries have consistently changed their habits during home confinement, which can also explain the increase in children's psychological and behavioral symptoms. Most children before quarantine used screens (e.g., tablets, TVs, mobiles, computers) <1 h. During home confinement, children are using screens for more than 3 h, which is definitely higher than levels recommended by international health organizations. For example, the WHO (28) suggests limiting screen time to 1 h for children under 6 years, and diverse studies have revealed associations between screen time and lower psychological well-being among children and adolescents (e.g., 28). In addition, physical activity was reduced in children given that greater than half now practice <30 min. However, before quarantine, children practiced between 30 and 60 min. This level is clearly below the WHO recommendations (28, 29) for at least 180 min of moderate to vigorous physical activity for children under 5 years and at least 60 min for children aged 5–17 years.

On the other hand, the results showed a positive change in children habits during quarantine, indicating an increase in the amount of sleep on weekdays. On average, children are sleeping 9.51 h per night (0.40 h more than before quarantine), which is more in accordance to WHO (28) and American Academy of Sleep Medicine (30) guidelines (10–13 h of good quality sleep for children under 5 years; 9–12 h for children 5–12 years; 8–10 h for adolescents). However, if this increase in sleep hours is associated with delays in bedtime (frequently related to the use of screen-based activities) (31), it could be problematic. This notion should be explored in future studies.

### Differences Across Italy, Spain, and Portugal

Considering differences of social distancing measures used in the three countries (i.e., mandatory quarantine in Italy and Spain vs. voluntary quarantine/duty of home confinement in Portugal), it was hypothesized that Italian and Spanish children would present higher psychological and behavioral symptoms associated with home confinement compared with Portuguese children. However, Italian children presented less symptoms of anxiety, as well as less sleep, feeding, and cognitive alterations compared with the other two groups of children. One possible explanation for this surprising result might be related to the fact that schools in Italy closed 8 days before schools in Spain and Portugal. Given that the data collection period occurred simultaneously in the three countries, the results may indicate that Italian children had more time to adjust to and accept the situation of home confinement and find strategies to cope with it (similar to a grief process) (32). Nevertheless, longitudinal studies are needed to explore this hypothesis. Another possible explanation might be associated with differences across countries in housing conditions and screen time. Greater than half of the Italian children live in homes with a garden, and these children spend less time on screens. On the other hand, although Portuguese children are living in larger houses, most Portuguese and Spanish children do not have outdoor exits, such as gardens or terraces, thus limiting available space for outdoor activities. Taken together, these results may suggest that Italian children are probably having more “quality play time” and engaging in activities that are healthier, screen free and in contact (although restricted) with nature compared with their counterparts, which can contribute to their reduced levels of psychological and behavioral symptoms during quarantine. These findings support studies showing children's positive outcomes associated with less screen time (33) and indicating that engagement in outdoor activities can play a protective role in terms of young people's mental health (34, 35).

Another important result pertains to Spanish children who presented more behavioral alterations. In particular, compared with Italians, Spanish children argue more with the rest of the family, have more behavioral problems, are angrier and are more dependent on their parents. These symptoms may be related to modifications in children's habits during quarantine given that this group exhibited a higher decrease of physical activity and higher levels of screen time. Previous studies have highlighted the consequences of oversedentary lifestyle and the use of screens by children and adolescents on their psychological well-being, including lower self-control, less emotional stability, more depressive and anxiety symptoms, and being more difficult to care for [e.g., (33, 36, 37)].

In turn, Portuguese children presented more mood alterations than Spanish children (e.g., feeling more lonely, sad, and frustrated) and are more children in this group feeling anxious and afraid of COVID-19 infection. This result is surprising considering that Portugal has adopted a less restrictive quarantine measure compared with the other countries in study. Some possible explanations may account for this finding. First, parental psychopathology (e.g., anxiety, depression) and offspring emotional disorders have been associated in the literature [e.g., (37, 38, 38–40)]. Consistent with this evidence, Portuguese adults present higher levels of psychiatric disorders compared with Spanish and Italian adults (22.9 vs. 9.2 and 8.2%, respectively; (41), and results showed high parental low mood and preoccupation with COVID-19, which seem to suggest the transmission of anxiety and distress from parents to children and/or within family relationships, contaminating children's mood. Second, these differences might also be related to the Portuguese quarantine status. It is possible that the non-mandatory nature of Portuguese home confinement may be confusing to children and adolescents in the sense that it might expose them to inconsistent situations of social contact (e.g., they might see children playing on the street while are told by their parents that they cannot do it). Future studies should explore these and other possible explanations for these results.

### Housing Conditions Predict Children's Psychological and Behavioral Symptoms During COVID-19 Quarantine

Our findings suggest that housing conditions, including having an outdoor exit such as a garden or terrace, and the number of people living at home, predicted children's psychological and behavioral symptomatology during COVID-19 quarantine. The results showed that having an outdoor exit in the house contributed to lower levels of all symptoms analyzed, except mood alterations. These findings are consistent with previous studies demonstrating that housing characteristics have a direct impact on people's well-being and mental health in general (42, 43), children's development and psychological health in particular [e.g., (44)], as well as young adults' mental health during COVID-19 lockdown (16). Evans et al. (15) argue that housing is not only a physical shelter but also a significant mental health and well-being resource. Having an outdoor exit might mean that children have more space to play freely in the house or direct contact with nature (in the case of houses with garden) and increases visual exposure and access to neighbors, thus elevating social contact (15). This feature is particularly important in a situation of home confinement and restriction of movements, promoting psychological well-being. In turn, not having these types of housing conditions may exacerbate the feeling of social isolation associated with being quarantined. This notion is consistent with evidence suggesting that families living in multiple-dwelling units experience more social isolation and lack of access to play spaces, thus keeping children inside apartment (15, 42). In accordance with previous evidence (15), the existence of possible underlying mechanisms that may account for the link between housing conditions and children's psychological symptoms but were not investigated in this study should be considered. Having more access to play space provided by outdoor exits may also promote more opportunities for parent-child positive interactions, facilitating more adequate parenting practices (less restrictive and less rigid control over children's activities) which is particularly important to foster children's psychological well-being in times of family stress such as COVID-19 quarantine.

Findings also showed that the number of people living at home contributed significantly to children's mood alterations. A possible explanation for this finding of the “the more the merrier” may be that in times of quarantine, where children have so few opportunities for social interaction and are deprived from contact with peers, interaction with siblings acquires an even greater importance in terms of psychological well-being. This relationship may act as a buffer against the stress caused by quarantine by providing playful interactions and a peer to whom to ask for help if needed, subsequently mitigating mood swings. This result adds to evidence demonstrating the importance of siblings relationships for children's and adolescents' well-being and mental health (45, 46).

Child sex and age also contributed significantly to children's psychological symptomatology. Being a boy predicted behavioral and feeding alterations, which is consistent with studies demonstrating that boys present higher levels of externalizing behaviors compared with girls (47). However, being a boy also predicted higher levels of anxiety symptoms, which is contrary to most of the studies that show higher prevalence of internalizing disorders among girls compared with boys (e.g., 46). Considering evidence demonstrating gender differences in physical activity levels, whereby boys are generally more physically active than girls (48, 49), these findings indicate that boys under quarantine are especially prone to develop diverse symptoms and behavioral alterations probably due to the restriction of movements and lack of opportunities to participate in physical activity that prevent boys from satisfying their developmental needs for physical activity. Actually, when compared with girls, boys presented higher levels of all studied symptoms. On the other hand, being younger also predicted behavior alterations as well as sleep changes, suggesting that younger children may be more vulnerable to the effects of home confinement. This finding is consistent with studies showing that young children are particularly vulnerable to stressful events (12) probably due to their needs of physical mobility and limited cognitive capability to understand the quarantine situation, as well as guidelines from international health organizations regarding young children responses to stress (50).

### Limitations and Implications for Future Studies

Some limitations of the present study should be mentioned. First, the cross-sectional nature of the study does not allow conclusions on cause-effect relationships, and the recruitment conducted through social networks with snowball sampling may have caused bias. Longitudinal studies and with representative samples will be crucial to deeply understand the real consequences of home confinement during COVID-19 pandemics. Second, this study relies on parent's perceptions about their children's psychological and behavioral alterations during quarantine. Parents' own level of distress may interfere with their perceptive capacities regarding children's functioning. In this sense, future studies should be based as much as possible on the report of the children and adolescents themselves regarding their home confinement experience. Because this experience and its consequences on mental health and well-being should differ according to the level of maturity of the individuals, future studies also need to compare different age groups in children and adolescents. Furthermore, it is necessary to identify and study other variables that may explain the higher levels of psychological and behavioral symptoms of children and adolescents (e.g., coping strategies, amount and quality of information about COVID-19) (17) given that the variance explained by hierarchical multiple regression models was very small. Future studies should also explore possible mediating variables related to parents or family environment (e.g., parental stress, number of hours working from home) that may diminish parental availability and attention as well as parental capacity to manage offspring difficulties and needs.

### Implications for Practice

It is essential that professionals and families are aware that being in home confinement is a hard, strange, and stressful situation for children and adolescents. It is expected that emotional and behavioral changes will occur as a way of expressing the difficulty in understanding, accepting, and adjusting to the situation. In addition, it is expected that most children return to their typical functioning provided that adequate routines and healthy habits are maintained during quarantine and children can receive consistent support from responsive caregivers (51, 52). However, some children may need psychological support after quarantine, especially those with previous psychological or development problems or those with parents struggling with mental problems or economic instability (51). To detect risk situations derived from the pandemic and home confinement (using validated multi-informant and multiproblem approaches), integrative intervention protocols are considered essential during and after home confinement (52).

Similarly, indicated preventive actions are absolutely crucial for the period after home confinement (e.g., at schools), which will allow the early detection of at-risk children, timely mitigation of the effects of a stressful situation for children and adolescents, and the reduction of mild symptoms before their aggravation (52).

Finally, because parents' and children's symptomatology are significantly related, specifically after pandemic disasters (10), and may have deleterious effects on parenting and family relationships, the identification of anxiety, depression, or posttraumatic stress disorder in children should lead clinicians to suggest screening parents' mental health.

### Recommendations for Families With Children and Adolescents

Emotional and behavioral changes are expected reactions in response to completely new situations for children and adolescents, such as being bored, lonely, irritable, uneasy, and worried, or having nightmares. It is therefore essential to pay particular attention to parenting practices at this stage, including adopting an even more authoritative discipline, talking to children about the situation using reliable and appropriate information for the child's age, and showing empathy with regard to their emotions, concerns, and frustrations for the losses that the pandemic is causing in their lives (e.g., being with friends, school routine, sport activities). It is important to set aside time to play with children, especially the smallest ones, increasing physical activity at home, for example, carried out with the family.

Children need structure, so it is essential to maintain rules and routines but also to create new “quarantine routines.” It could be helpful create a flexible but consistent daily routine, including time for schoolwork and chores in which children could participate, a specific bedtime and wake-up time, and playtime with and without the family. It is important that children and adolescents can use their phone to connect with friends, compensating for the absence of face-to-face interactions. However, they should also have technology-free time.

To compensate for the lack of space for outdoor activities (especially for those who do not have houses with gardens or terraces), it is important to engage in family activities and games that increase positive parent-child interactions and physical activity and consequently avoid excessive screen-time increases. As stated by Wang et al. (17), “with the right parenting approaches, family bonds can be strengthened, and child psychological needs met.”

Finally, it is also important that parents also monitor their own behavior and adopt self-care behaviors given that children's and adolescent's adaptation and coping with this situation is largely mediated by the role of parents and other relevant social agents (52).

## Conclusion

Our study contributes to an emergent body of literature regarding the adverse psychological outcomes associated with COVID-19 quarantine on children and adolescents, consequences that remain uncertain in a population still understudied in the field of pandemic research. The psychological stress as well as individual and family patterns' alterations imposed by home confinement interact with housing conditions, contributing to detrimental effects on children's and adolescents' physical and mental health. Primary and secondary prevention measures are urgently needed to mitigate these effects; otherwise, they can be long lasting and negatively influence youth development.

## Data Availability Statement

The raw data supporting the conclusions of this article will be made available by the authors, without undue reservation.

## Ethics Statement

The studies involving human participants were reviewed and approved by Ethics Committee of Católica Research Centre for Psychological, Family and Social Wellbeing; Ethics Committee of Miguel Hernandez University; Ethics Committee of Università degli Studi di Perugia. The patients/participants provided their written informed consent to participate in this study.

## Author Contributions

RF designed the Portuguese survey, collected data, and wrote the draft of this article. MP collected data and wrote the draft of this article. ED designed the Italian survey and collected data. JE designed the study. AM managed and analyzed data. CM collected data. MO designed the study and the survey. All the authors reviewed the draft and contributed to the final version of the manuscript.

## Conflict of Interest

The authors declare that the research was conducted in the absence of any commercial or financial relationships that could be construed as a potential conflict of interest.
